# Role of baseline soluble tumor necrosis factor receptor 2 as a biomarker in primary podocytopathy: Implications for renal impairment and disease progression

**DOI:** 10.1186/s12882-024-03772-y

**Published:** 2024-10-25

**Authors:** Srinivas Nagaram, Priscilla Charles, Yadav Nisha, Norton Stephen, Nandeesha Hanumanthappa, Sreejith Parameswaran, Palanivel Chinnakali, Rajesh Nachiappa Ganesh

**Affiliations:** 1 Department of Pathology, Jawaharlal Institute of Postgraduate Medical Education and Research, Puducherry, 605006 India; 2 Department of Medical Oncology, Jawaharlal Institute of Postgraduate Medical Education and Research, Puducherry, 605006 India; 3 Department of Biochemistry, Jawaharlal Institute of Postgraduate Medical Education and Research, Puducherry, 605006 India; 4 Department of Nephrology, Jawaharlal Institute of Postgraduate Medical Education and Research, Puducherry, 605006 India; 5 Department of Preventive and Social Medicine, Jawaharlal Institute of Postgraduate Medical Education and Research, Puducherry, 605006 India

**Keywords:** Biomarker, Tumor necrosis factor alpha, Podocytopathies, Estimated glomerular filtration rate, Chronic kidney disease

## Abstract

**Background:**

Podocytopathies, including minimal change disease (MCD), focal segmental glomerulosclerosis (FSGS), and collapsing glomerulopathy (CG), are kidney diseases that damage glomerular podocytes, leading to heavy proteinuria and nephrotic syndrome (NS). Inflammation plays a critical role in the progression of chronic kidney disease (CKD), with recent studies linking inflammatory biomarkers to declining kidney function. Tumor necrosis factor-alpha (TNF-α), an essential inflammatory cytokine, interacts with its circulating receptors, TNFR1 and TNFR2. The TNF-α pathway has been implicated in the pathogenesis of FSGS and MCD. Increased circulating TNFR2 levels have been associated with worsening renal function in podocytopathies, suggesting that the TNF-α inflammatory pathway significantly contributes to disease progression.

**Methods:**

We conducted a study involving 53 patients with biopsy-proven MCD or FSGS and 53 healthy, age- and gender-matched controls. All patients were followed for 18 months. We analyzed serum and urine TNFR2 levels and gene expression at baseline and after three months. To assess the ability of TNFR2 to predict persistent decline in estimated glomerular filtration rate (eGFR < 30 mL/min/1.73m^2^), remission, and relapse, we employed Cox regression analysis. Additionally, we evaluated its prognostic utility for predicting progression to stage 4 CKD using ROC curve analysis.

**Results:**

Serum and urine TNFR2 levels were significantly elevated in patients compared to controls. Serum TNFR2 was a significant predictor in univariate Cox regression analysis for persistent eGFR decline (HR 1.017, 95% CI: 1.003 to 1.032, *p* = 0.018), remission (HR 0.995, 95% CI: 0.992 to 0.999, *p* = 0.006), and relapse (HR 1.005, 95% CI: 1.001 to 1.010, *p* = 0.029). The ROC curve analysis demonstrated that serum TNFR2 levels had a strong prognostic ability for predicting progression to stage 4 CKD, with an AUC of 0.848 (95% CI: 0.737—0.960), sensitivity of 81%, and specificity of 71%.

**Conclusion:**

This study underscores the critical role of circulating TNFR2 in kidney injury among patients with primary podocytopathy. Elevated TNFR2 levels are significant predictors of persistent eGFR decline and disease relapse, highlighting their potential as biomarkers for disease progression and prognosis.

**Supplementary Information:**

The online version contains supplementary material available at 10.1186/s12882-024-03772-y.

## Introduction

Podocytopathies refer to a group of renal diseases characterized by heavy proteinuria and nephrotic syndrome (NS) caused by diffuse effacement of podocytes in most of the glomerular capillary loops on ultrastructural examination in a setting where no immune complex deposits are identified. Minimal change disease (MCD), focal segmental glomerulosclerosis (FSGS), and collapsing glomerulopathy (CG) are characteristic podocytopathies [1, 2]. The pathogenesis underlying MCD and FSGS is multifaceted, and involves genetic variations, epigenetic factors, immunological responses, metabolic influences, and viral components that may contribute to the development of these diseases [3]. Recent studies have highlighted the critical role of the actin cytoskeleton in maintaining podocyte structure and function. Disruptions in the actin cytoskeleton can lead to podocyte effacement, a hallmark of diseases like MCD and FSGS, thereby compromising the glomerular filtration barrier [4]. This cytoskeletal disruption is a key contributor to podocyte injury, which underscores the importance of identifying early biomarkers for podocyte damage and disease progression. T-regulatory cell dysfunction and abnormalities in the podocyte proteins CD80/ angiopoietin-like protein 4 (Angptl4) have been associated with the development of MCD. It is believed that either the immune system or a genetic defect plays a role in the onset of primary FSGS and CG. Both MCD and FSGS are frequent contributors to NS, which is characterized by significant proteinuria, edema, and intravascular volume depletion [1].

Primary podocytopathies are not associated with immune complex deposition in renal cortex and the resultant inflammatory cell infiltration. Thus the immune related pathway is not widely studied in disease etio-pathogenesis and progression of primary podocytopathies. We conducted a comprehensive literature review using PubMed and Scopus databases on the role of TNFR2 as a biomarker in podocytopathies focusing on publications from 2000 to 2024. The search centered on TNFR2 and its role in kidney diseases, specifically podocytopathies, to identify gaps in the current understanding and establish a basis for the design of our study.

MCD is a common glomerular disease among children and a significant cause of primary NS in adults, accounting for 10 to 15% of cases. Conversely, FSGS is more commonly diagnosed in adults, accounting for approximately 40% of adult cases and 20% of pediatric cases [3, 5]. FSGS is characterized by steroid-resistant nephrotic syndrome unlike MCD, and podocyte damage progressively leads to adhesions between the exposed capillary and Bowman’s capsule and sclerosis of the glomerular capillary segment. A significant number of FSGS patients progress to end-stage renal failure, requiring either dialysis or kidney transplantation [6]. In recent years, several studies have highlighted the role of TNFR2 and TNF-α in kidney disease progression. For instance, Gohda et al. demonstrated the correlation between elevated TNFR2 levels and reduced eGFR in patients with diabetes [7], while the NEPTUNE study revealed similar findings in patients with FSGS [8]. Our study aims to build on these findings by evaluating TNFR2’s prognostic utility specifically in podocytopathies, an area where limited data currently exists.

Regardless of etiology and pathogenesis, inflammation plays a vital role in the renal disease progression towards CKD. Recent research highlights a correlation between inflammatory biomarkers and a decline in kidney function, shedding light on the underlying mechanisms of kidney injury [9]. Tumor necrosis factor-alpha (TNF-α) is a key proinflammatory and immunoregulatory mediator, produced by macrophages and various intrinsic kidney cells including podocytes, mesangial cells, epithelial cells, and endothelial cells [10, 11]. Both circulating free TNF-α and TNF-α bound to circulating tumor necrosis factor receptors (TNFRs), specifically TNFR1 and TNFR2, are detected in plasma [12]. Elevated levels of circulating TNFRs have been observed in individuals with CKD [10]. The involvement of the TNF-α pathway seems to be significant in the development of FSGS and MCD [6, 13]. Specifically, elevated levels of circulating TNFR2 have been linked to a reduction in renal function, indicating that the TNF-α inflammatory pathway may mirror disease progression [9]. TNF receptors serve not only as potential biomarkers of renal damage but also as active mediators of these diseases [14].

Research indicates a positive correlation between circulating TNF-α receptor levels and urinary albumin-to-creatinine ratio (ACR), and a negative correlation with the glomerular filtration rate (eGFR) in patients with various renal disorders [7]. Moreover, higher levels of soluble TNFR2 (sTNFR2) are independently associated with accelerated rates of decline in kidney function resulting in CKD [15]. Elevated levels of circulating TNFRs at diagnosis have shown promise as effective predictors of renal progression. The precise mechanisms linking elevated circulating TNFR levels with an increased risk of renal progression are not yet fully understood [16]. The aim of this study was to evaluate the ability of TNFR2 to predict persistent decline in the eGFR, its association with remission and relapse, and its correlation with biochemical parameters and histopathological features in patients diagnosed with podocytopathy.

## Materials and methods

### Study design

The aim of this research was to investigate the levels of TNFR2 in blood and urine, as well as its gene and renal expression, and demonstrate its predictive and prognostic significance for persistent eGFR decline, relapse, and remission among biopsy proven patients with primary podocytopathy. The research was conducted in the Department of Pathology at the Jawaharlal Institute of Medical Education and Research and received approval from the institute’s ethics committee for human studies (JIP/IEC/2019/070). This study adhered to the ethical principles specified in the Helsinki Declaration.

### Characteristics of the study participants

After obtaining consent, we enrolled 53 newly diagnosed NS patients with biopsy-proven primary podocytopathy (52.83% with MCD and 47.16% with FSGS) from the Nephrology Department at JIPMER. Patients with obvious malignancies, other immune complex mediated glomerulonephritis, such as lupus, and those using incriminating drugs were excluded. We followed all the patients for a minimum of 18 months, with 3 monthly monitoring of urine protein, serum creatinine, lipid profile and blood pressure. Baseline clinical data were recorded, and we collected blood and urine samples at the time of initial biopsy and at the 3- month follow-up. Blood samples were centrifuged and stored at -80 °C, and RNA was isolated from blood from EDTA tubes. We calculated the eGFR using the CKD-EPI creatinine 2009 equation and defined remission and relapse based on KDIGO guidelines [17]. Additionally, we collected blood and urine samples from age and sex-matched healthy controls for comparison.

The cutoff for defining significant kidney function decline was set at an eGFR < 30 mL/min/1.73 m^2^. This value corresponds to stage 4 CKD, which is a critical threshold in the management decision for initiation of renal replacement therapy for dialysis or renal transplantation. However, in individual patients, the decision to start a patient on dialysis or initiate renal transplantation proceedings are affected by several factors such as co-existing infections, availability of vessels for arterio-venous shunt, suitable donors and financial constraints etc. Hence, we decided to stick to the clinically significant threshold of eGFR of < 30 mL/min/1.73 m^2^ uniformly in all patients to avoid additional confounders.

### ELISA

Serum and urine samples were subjected to protein quantification using enzyme-linked immunosorbent assay (ELISA) kits for TNFR2 (KTE60215, Abbkine, Wuhan, China) and TNF-α (KTE6032, Abbkine, Wuhan, China). The sandwich ELISA technique was used, with 10 µl of sample for TNFR2 and 100 µl for TNF-α. Protein levels were measured using a Bio-Rad Immunowash 1575 washer and a microplate reader (Bio-Rad, California, United States) set to 450 nm within 15 min of sample preparation. Protein concentrations were determined based on standard curves provided by the respective kits.

### Gene expression

RNA was isolated from peripheral blood using the QIAmp Blood RNA Mini Kit (52304, QIAGEN, Hilden, Germany) and was purified and assessed for purity using the Qubit_TM RNA HS Kit (Q32852, Invitrogen by Thermo Fisher Scientific, United States) with a Qubit 3.0 fluorometer (Q33216, Invitrogen by Thermo- Fisher Scientific, United States) to ensure the absence of protein and DNA contamination. A high-capacity cDNA conversion kit (4368814, Applied Biosystems by Thermo Fisher Scientific, United States) was used to synthesize complementary DNA (cDNA) from contaminant-free RNA. cDNA was amplified and quantified using an Applied Biosystems Quantstudio-3 thermal cycler and TaqMan fast advanced PCR master mix (4444963, Applied Biosystems by Thermo Fisher Scientific, United States) with TaqMan probes specific for TNFR2 (Hs00153550-m1, Applied Biosystems by Thermo Fisher Scientific, United States) and the housekeeping gene GAPDH (Hs99999905-m1, Applied Biosystems by Thermo Fisher Scientific, United States). PCR amplification involved specific temperature cycles, and relative quantification was determined using the 2^-ΔΔCt formula [18].

### Histopathological assessment of renal abnormalities

Glomerulosclerosis (GS), both global and segmental, was determined as the percentage of globally or segmentally sclerosed glomeruli in the initial biopsy and categorized using the same four-point scale. Tubular atrophy (TA) and interstitial fibrosis (IF) were evaluated semi-quantitatively using a four-point scale: absent (< 10%—score 0), mild (10–25%—score 1), moderate (26–50%—score 2), and severe (≥ 50%—score 3) of the total cortical area [19].

### Immunohistochemical analysis

Formalin-fixed paraffin sections of renal tissue were cut to 3–4 µm thickness and subjected to deparaffinization, rehydration, and endogenous peroxidase activity inhibition using 5% hydrogen peroxide. Antigen retrieval was achieved with citrate buffer in a pressure cooker, followed by washing with Tris-buffered saline (TBS). The slides were then incubated overnight at 4 °C with a rabbit primary antibody against TNFR2 (ab109322) at a dilution of 1:400. After incubation, the slides were washed with TBS and incubated with a HRP-conjugated secondary antibody for 40 min. Visualization of antigen–antibody complexes was performed using DAB chromogen for 5 min, followed by counterstaining with Harris Hematoxylin. The H-scoring system was used to assess marker expression in podocytes, proximal convoluted tubules (PCT), and distal convoluted tubules (DCT) based on proportion and intensity. The study of proximal and distal tubules are very characterized in histopathological examination of renal biopsies and all the findings were interpreted by experienced renal Pathologists. In addition, each of the immunohistochemical image is analyzed in correlation with biopsy slides stained with hematoxylin and eosin (H&E), Periodic acid Schiff (PAS), Gomori methenamine silver with PAS and Masson trichrome stains and the distinction of proximal and distal convoluted tubules in immunohistochemistry is also correlated with findings of brush border, and abundant cytoplasm in PAS stains.

#### Statistical analysis

The data distribution was assessed using the Shapiro–Wilk test. Continuous variables are summarized as the mean ± standard deviation (SD) or median with interquartile range (IQR), while categorical variables are presented as percentages. Bivariate Spearman rank correlation was used to analyze the relationships between baseline serum and urine TNFR2 levels and various other factors. The Wilcoxon signed-rank test is employed to compare the baseline and follow-up groups, and the Mann–Whitney U-test was utilized to compare the cases to healthy controls. Kaplan–Meier analysis with a log-rank test was used to evaluate time-to-event, while Cox regression was used to evaluate predictive biomarkers for eGFR decline in patients with CKD stage 4 and relapse. A receiver operating characteristic ROC curve was applied to validate the predictive utility for persistent eGFR decline below CKD stage 4. The Kruskal–Wallis test was performed to assess the association between baseline serum TNFR2 levels and histopathological chronicity scores. SPSS version 19 and GraphPad Prism version 8.0.2 were used, with *p* < 0.050 indicating statistical significance.

## Results

**Fig. 1:**
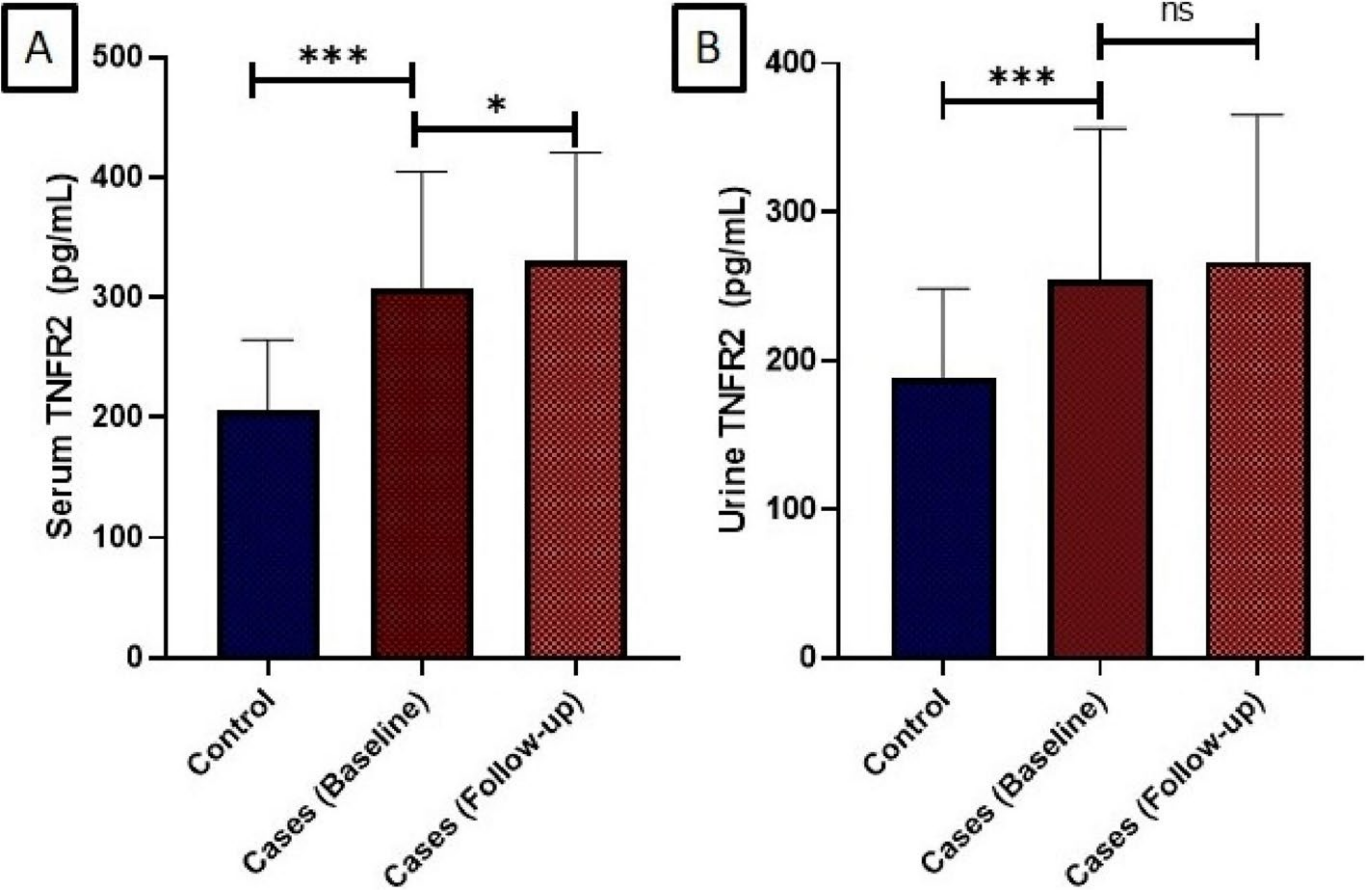
Comparing TNFR2 levels in cases at baseline, follow-up (**A**), and versus controls (**B**). Comparing TNFR2 levels between baseline and follow-up in podocytopathy patients (**A**) and comparing patients versus controls (**B**). The figure shows the significant elevation of TNFR2 levels in patients and in comparison to healthy controls, emphasizing its potential role as a biomarker for disease progression. TNFR2, Tumor necrosis factor receptor 2

The baseline demographic and biochemical characteristics of the study participants were presented based on the median TNFR2 level (Table 1). The median serum TNFR2 levels in patients with primary podocytopathy were 305.30 pg/mL (IQR: 217.27—399.01 pg/mL) at baseline and 356.75 pg/mL (IQR: 253.13—402.70 pg/mL) at follow-up. In healthy controls, it was 205.20 pg/mL (IQR: 150.16—249.37 pg/mL). The median urine TNFR2 levels were 252.77 pg/mL (IQR: 173.82—356.43 pg/mL) at baseline and 274.20 pg/mL (IQR: 181.60—360.90 pg/mL) at follow-up. In healthy controls, it was 164.20 pg/mL (IQR: 139.54—245.53 pg/mL) (Fig. 1A, B).

**Table 1:** Clinical characteristics of podocytopathy patients according to median serum TNFR2 level at baseline

Parameter	Total	Above Median (> 305.30)	Below Median (< 305.30)	*p*-value
Age	30.4 ± 9.8	32.6 ± 9.7	28.1 ± 9.9	0.106
MAP (mm Hg)	91.98 ± 8.8	91.29 ± 9.58	93.17 ± 1.53	0.367
eGFR (mL/min/1.73 m^2^)	120 (99 – 135)	102 (76 – 131)	128 (118 – 137)	0.001
UPCR (mg/mg)	0.86 (0.11 – 8.80)	6.3 (1.88 – 10.66)	0.29 (0.11 – 1.42)	0.001
TNF—α (pg/mL)	6.5 (4.90 – 14.61)	14.40 (7.52 – 17.43)	5.15 (4.30 – 6.42)	0.001
Serum albumin (gm/dL)	3.15 ± 1.11	2.53 ± 0.92	3.79 ± 0.20	0.001
Total Protein (gm/dl)	6.05 ± 1.09	5.44 ± 0.19	6.61 ± 0.16	0.001
Blood Glucose (mg/dL)	89.45 (83.63 – 104.47)	90.81 (84.70 – 98.88)	86.85 (82.46 – 105.62)	0.620
Blood Urea (mg/dL)	24.33 (19.83 – 28.64)	27.33 (20.01 – 43.28)	22.08 (18.25 – 26.56)	0.003
Sodium (mEq/L)	137.03 ± 2.21	136.37 ± 2.75	137.63 ± 1.34	0.079
Potassium (mEq/L)	4.07 ± 0.32	4.02 ± 0.35	4.13 ± 0.05	0.149
Calcium (mg/dL)	8.91 ± 0.77	8.65 ± 0.85	9.17 ± 0.60	0.022
Magnesium (mg/dL)	1.88 ± 0.24	1.78 ± 0.19	1.96 ± 0.05	0.034
Phosphorus (mg/dL)	3.92 ± 0.49	3.94 ± 0.50	3.93 ± 0.53	0.988
Chloride (mEq/L)	100.98 ± 7.43	102.81 ± 3.10	98.55 ± 10.30	0.082
Uric acid (mg/dL)	5.86 ± 1.63	5.71 ± 1.53	6.07 ± 1.72	0.458
Total Bilirubin (mg/dL)	0.45 (0.34 – 0.58)	0.42 (0.31 – 0.57)	0.50 (0.40 – 0.63)	0.173
Direct Bilirubin (mg/dL)	0.09 (0.06 – 0.14)	0.09 (0.06 – 0.13)	0.09 (0.06 – 0.15)	0.939
AST (IU/L)	20.67 (17.50 – 25.10)	18.50 (19.25 – 22.54)	21.25 (19.20 – 26.73)	0.039
ALT (IU/L)	18.21 (14.85 – 22.63)	19.10 (13.16 – 21.70)	18. 21 (15.32 – 23.05)	0.249
ALP (IU/L)	83.67 (74.67 – 102.22)	82.33 (69.87 – 97.1)	89.25 (77.75 – 123.2)	0.145
Total Cholesterol(mg/dL)	251.1 (184.5 – 309.3)	300.3 (201.2 – 477.5)	240.5 (168.5 – 265.1)	0.088
Hemoglobin (gm/dL)	12.66 ± 2.06	11.85 ± 1.98	13.71 ± 1.59	0.001
WBC count (cells/μL)	10.92 (9.23 – 13.69)	11.65 (9.21 – 13.83)	10.52 (9.26 – 12.41)	0.510

*TNFR2* Tumor necrosis factor receptor 2, *MAP* Mean arterial pressure, *eGFR* Estimated glomerular filtration Rate, *UPCR* Urine protein-to-creatinine ratio, *TNF-α* Tumor necrosis factor-alpha, *AST* Aspartate aminotransferase, *ALT* alanine aminotransferase, *ALP* alkaline Phosphatase, *WBC* White blood cell count

We found serum TNFR2 levels were positively correlated with baseline UPCR (Urine Protein-to-Creatinine Ratio) (*r* = 0.788, *p* < 0.001), serum TNF-α (*r* = 0.662, *p* < 0.001), total cholesterol (*r* = 0.540, *p* < 0.001), and negatively correlated with eGFR (*r* = -0.371, *p* = 0.007), serum albumin (*r* = -0.681, *p* < 0.001), and calcium (*r* = -0.479, *p* < 0.001). Similarly, urine TNFR2 levels are positively correlated with baseline UPCR (*r* = 0.806, *p* < 0.001), TNF-α (*r* = 0.638, *p* < 0.001), total cholesterol (*r* = 0.499, *p* < 0.001), and negatively correlated with serum albumin (*r* = -0.552, *p* < 0.001) and serum calcium (*r* = -0.365, *p* = 0.008) (Table 2).

**Table 2:** Correlation of serum TNFR2 levels with age, MAP and other baseline biochemical parameters

		**Age**	**MAP**	**eGFR**	**UPCR**	**TNF- α**	**SA**	**Ca**	***P***	**UA**	**TC**
**Serum TNFR2**	r	.212	.024	**-.371**	**.788**	**.662**	**-.681**	**-.479**	.144	-.171	**.540**
	*p* –value	.128	.866	**.007**	**0.001**	**0.001**	**0.001**	**0.001**	.502	.226	**0.001**
**Age**	r	1.000	-.004	-.549	.134	.098	-.054	-.106	-.042	-.279	.228
	*p* -value		.977	.000	.398	.483	.711	.449	.844	.045	.164
**MAP**	r		1.000	-.047	.097	-.039	.107	.121	-.209	.150	.012
	*p* -value			.745	.543	.782	.463	.387	.327	.289	.940
**eGFR**	r			1.000	-.176	-.350	.227	.168	-.271	.052	-.162
	*p* -value				.270	.012	.121	.237	.201	.722	.339
**UPCR**	r				1.000	.698	-.738	-.390	-.031	-.190	.569
	*p* -value					0.001	0.001	.011	.891	.235	.001
**TNF- α**	r					1.000	-.592	-.399	.126	-.097	.420
	*p* -value						0.001	.003	.558	.493	.008
**SA**	r						1.000	.464	-.170	.089	-.316
	*p* -value							.001	.451	.542	.060
**Ca**	r							1.000	-.119	.089	-.671
	*p* -value								.580	.529	.000
**P**	r								1.000	.026	-.375
	*p* -value									.903	.138
**UA**	r									1.000	-.162
	*p* -value										.324

Serum TNFR2 levels were positively correlated with UPCR, serum TNF-α, and total cholesterol, while negatively correlated with eGFR, serum albumin, and calcium. All correlations were statistically significant (*p* < 0.05)

*MAP* Mean arterial pressure, *eGFR* Estimated glomerular filtration Rate, *UPCR* urine protein creatinine ratio, *TNF-α* tumor necrosis factor-alpha, *SA* serum albumin, *Ca* calcium, *eGFR* estimated glomerular filtration rate, *MAP* mean arterial pressure, *P* phosphorus, *TC* total cholesterol, *UA* uric acid

**Fig. 2:**
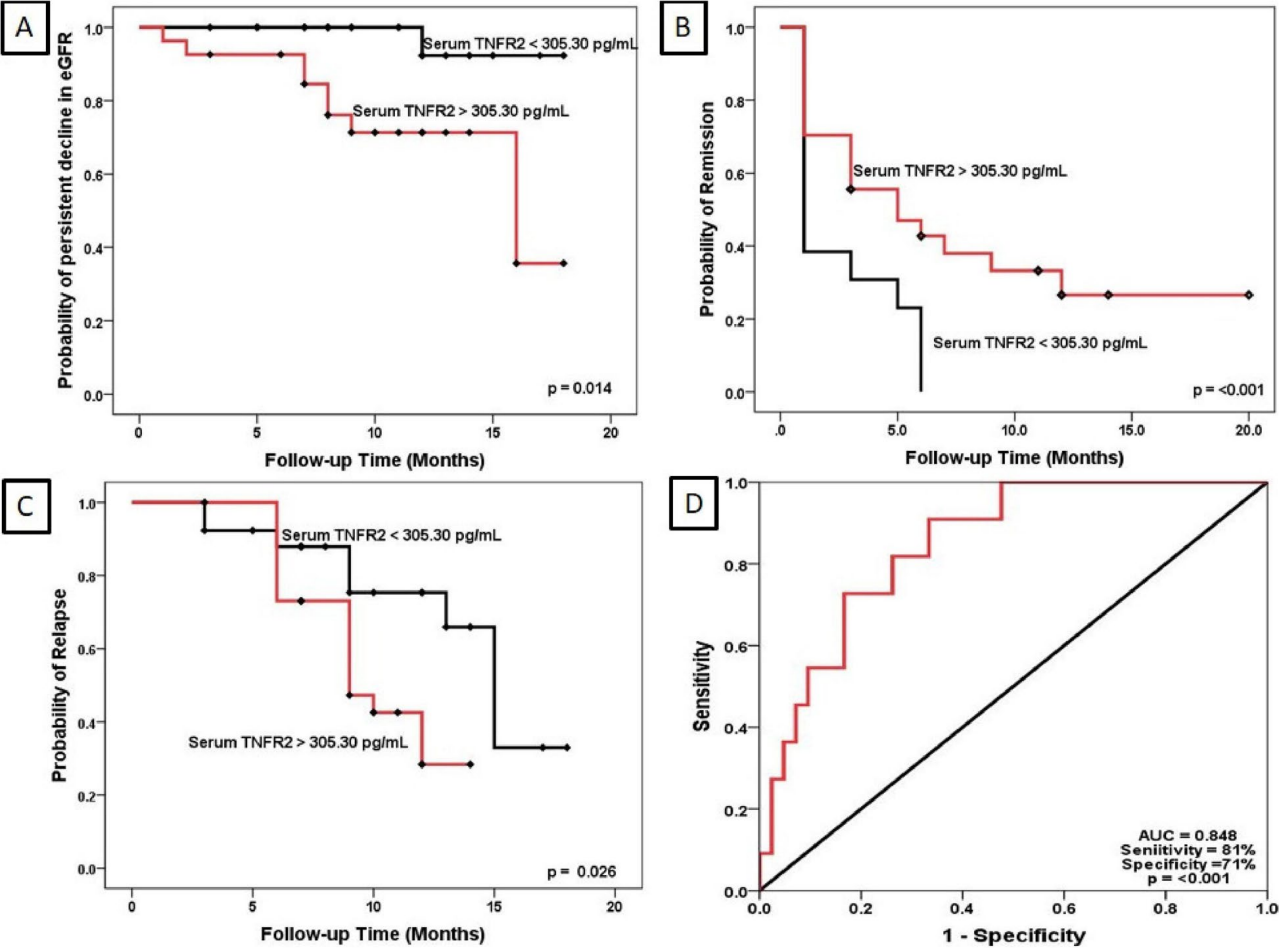
Kaplan–Meier for eGFR decline (**A**), remission (**B**), relapse (**C**), stratified by TNFR2 and ROC for predictive utility (**D**). Kaplan–Meier curves for estimating eGFR decline (**A**), remission (**B**), and relapse (**C**) stratified by TNFR2 levels. (**D**) represents the Receiver Operating Characteristic (ROC) curve demonstrating the predictive utility of TNFR2 for persistent eGFR decline, with an area under the curve (AUC) highlighting sensitivity and specificity. TNFR2, Tumor necrosis factor receptor 2; eGFR, Estimated glomerular filtration rate; ROC, Receiver operating characteristic; AUC, Area under the curve

We examined the effects of circulating TNFRs on progressive decline of renal function, stratifying the eGFR levels at the time of diagnosis to evaluate for persistent decline of eGFR below < 30 mL/min/1.73m^2^ during follow-up. Podocytopathy patients with serum TNFR2 below median levels at presentation did not experience a decline in eGFR below stage 4 CKD on follow-up (*p* = 0.014). These patients with below-median serum TNFR2 levels at presentation had earlier remission (*p* < 0.001) and lower probability of relapse (*p* = 0.026) on follow-up (Fig. 2A-C). Additionally, we performed Cox regression analysis to assess the potential predictors of persistent decline in eGFR, remission and relapse (Table 3). In the univariate Cox regression analysis, serum albumin (HR 0.491, 95% CI: 1.25 to 0.94, *p* = 0.033), serum TNFR2 (HR 1.017, 95% CI: 1.003 to 1.032, *p* = 0.018), and serum TNF-α (HR 1.114, 95% CI: 1.00 to 1.23, *p* = 0.038) were found to be significant predictors of persistent decline in eGFR. For predicting remission, only serum TNFR2 (HR 0.995, 95% CI: 0.992 to 0.999, *p* = 0.006) was found to be significant in the univariate Cox regression analysis. Serum albumin (HR 0.633, 95% CI: 0.41 to 0.95, *p* = 0.29), and serum TNFR2 (HR 0.1005, 95% CI: 1.001 to 1.010, *p* = 0.29) were found to be significant predictors of relapse in the univariate Cox regression analysis.

**Table 3:** Cox regression analysis for factors predicting eGFR decline, remission, and relapse in podocytopathy

	**Persistent decline in eGFR(< 30 mL/min/1.73 m**^2^**)**			**Remission**			**Relapse**		
	**HR**	**95% CI**	***p*-value**	**HR**	**95% CI**	***p*-value**	**HR**	**95% CI**	***p*-value**
**Univariate**									
Age	1.046	0.99 – 1.09	0.059	0.987	0.95 – 1.01	0.395	1.020	0.98 – 1.05	0.311
Sex	1.552	0.43 – 5.50	0.496	0.602	0.32 – 1.11	0.108	1.443	0.64 – 3.22	0.372
MAP	1.029	0.95 – 1.10	0.423	0.992	0.95 – 1.03	0.661	1.019	0.97 – 1.07	0.448
HTN	0.176	0.021 – 1.47	0.109	1.970	0.27 – 14.35	0.503	0.664	0.08 – 4.98	0.690
DM	0.953	0.12 – 7.51	0.963	0.648	0.19 – 2.14	0.477	2.477	0.33 – 18.62	0.378
Serum albumin	0.491	0.25 – 0.94	**0.033**	1.267	0.94 – 1.70	0.120	0.633	0.41 – 0.95	**0.029**
Serum Calcium	0.621	0.30 – 1.27	0.192	1.313	0.85 – 2.06	0.217	0.733	0.42 – 1.25	0.257
Serum Phosphorus	1.525	0.55 – 4.17	0.412	0.736	0.42 – 1.27	0.274	0.708	0.34 – 1.47	0.355
Serum TNFR2	1.017	1.003 – 1.032	**0.018**	0.995	0.992– 0.999	**0.006**	1.005	1.001 – 1.010	**0.029**
Serum TNF-α	1.114	1.00 – 1.23	**0.038**	0.943	0.88 – 1.00	0.062	1.075	0.99 – 1.16	0.066
**Multivariable**									
Serum albumin	0.569	0.24 – 1.35	0.202	–	–	–	0.748	0.43 – 1.28	0.291
Serum TNFR2	1.004	1.000 – 1.008	0.084	–	–	–	1.003	0.99 – 1.00	0.346
Serum TNF-α	1.000	0.86 – 1.15	0.997	–	–	–	–	–	–

Receiver operating characteristic (ROC) curve analysis was performed to calculate the area under the curve (AUC) for serum TNFR2, determining its prognostic ability to predict progression to stage 4 CKD during follow-up. The ROC curve showed an AUC of 0.848 (95% CI: 0.737—0.960), with a sensitivity of 81% and specificity of 71% for predicting progression to stage 4 CKD (Fig. 2D). We performed linear regression to assess the association between serum TNFRs and the eGFR slope. TNFR2 showed a positive but non-significant association (β = 0.508, *p* = 0.092), while TNF-α was significantly correlated with the eGFR slope (β = 0.562, *p* = 0.049). For doubling of serum creatinine, we used logistic regression, which identified significant associations with serum albumin (Exp (B) = 0.008, *p* = 0.019) and UPCR (Exp (B) = 0.383, *p* = 0.034). TNFR2 showed a non-significant trend towards association (Exp (B) = 1.026, *p* = 0.090).

**Fig. 3:**
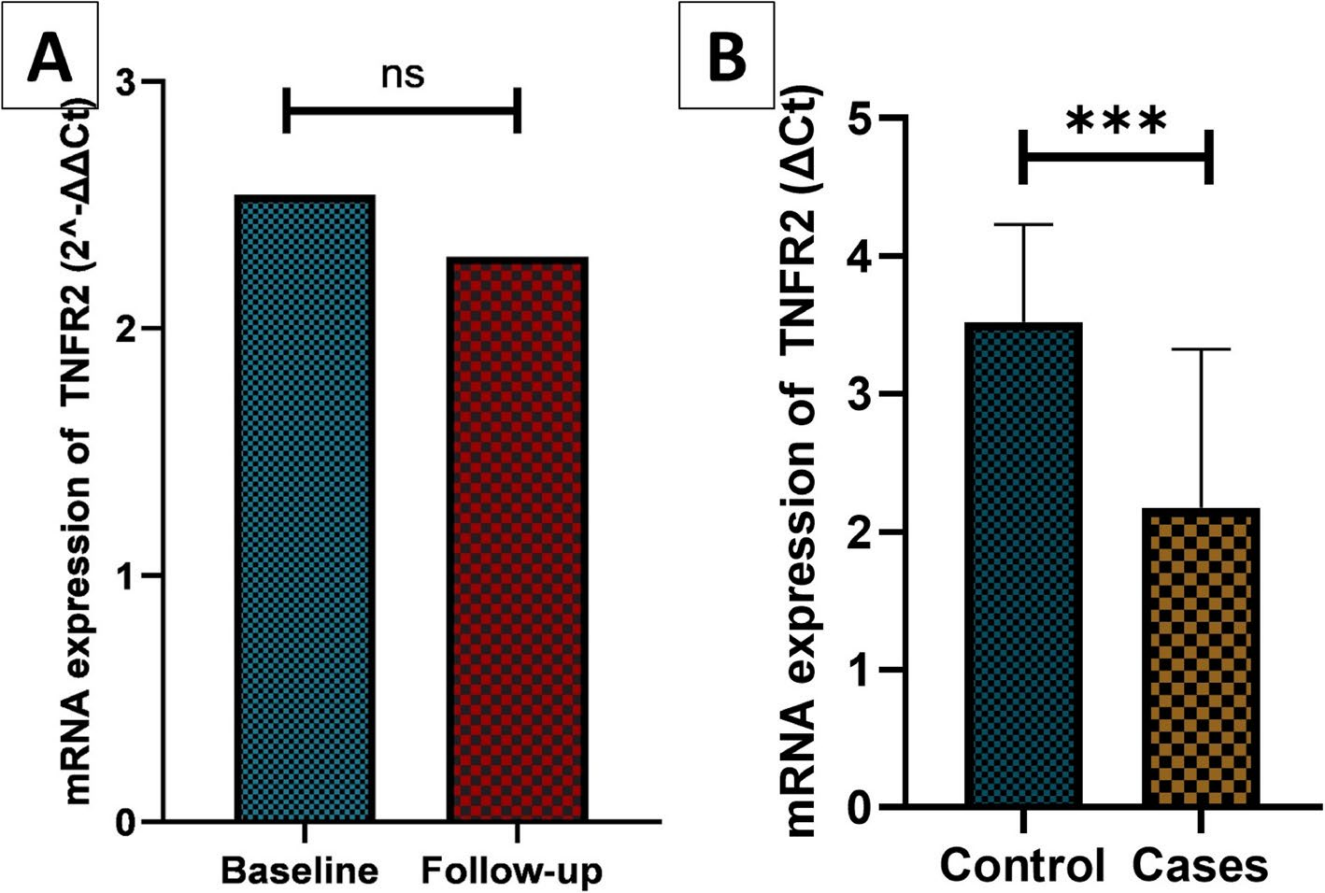
Comparison of TNFR2 gene expression between cases at baseline and follow-up (**A**) and between cases and controls (**B**). TNFR2 gene expression comparison between baseline and follow-up in patients with podocytopathy (**A**) and between patients and controls (**B**). The figure highlights significant upregulation of TNFR2 mRNA in cases, reinforcing its potential as a disease marker. sTNFR2, Tumor necrosis factor receptor 2, mRNA: Messenger ribonucleic acid

**Fig. 4:**
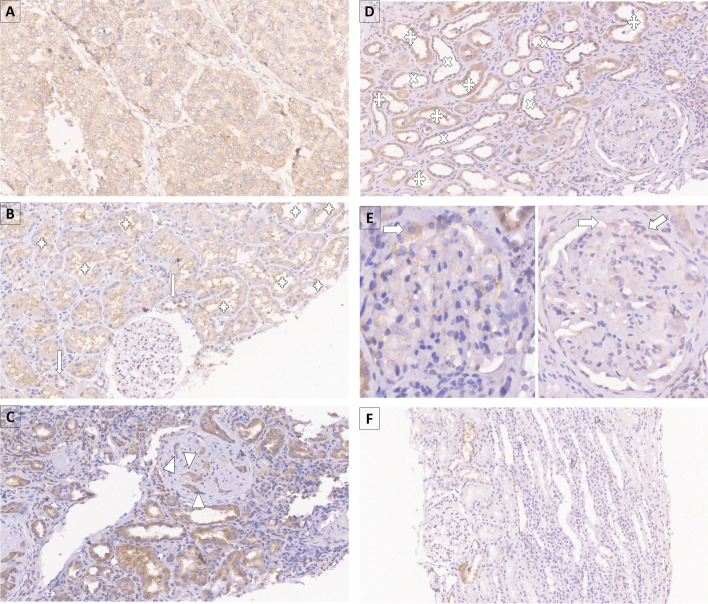
Renal expression of TNFR2 through immunohistochemical (IHC) staining. **A** Section shows positive control for TNFR2 in invasive tumor cells of invasive breast carcinoma of no special type. Diaminobenzidine stain, Immunoperoxidase stain with TNFR2, X400. **B** Section shows weak focal staining for TNFR2 in Mesangial Cells, and weak diffuse staining in proximal and distal convoluted tubules in kidney biopsy of a patient with minimal change disease, Diaminobenzidine stain, Immunoperoxidase stain with TNFR2, X400.  Proximal convoluted tubules and  Distal convoluted Tubules. **C** Section shows strong diffuse staining in non-atrophic proximal and distal convoluted tubules and mesangial cells in glomeruli in kidney biopsy of a patient with focal segmental glomerulosclerosis. The atrophic segment of tubules and scarred focus of glomerulus are negative for TNFR2. Diaminobenzidine stain, Immunoperoxidase stain with TNFR2, X400.  Mesangial cells being highlighted by TNFR2 immunohistochemistry staining. **D** Section shows strong diffuse staining in proximal and distal convoluted tubules and focal weak staining in podocytes in kidney biopsy of a patient with focal segmental glomerulosclerosis. Diaminobenzidine stain, Immunoperoxidase stain with TNFR2, X400.  Highlights distal convoluted tubules and  indicates proximal convoluted tubules. **E** Sections show two glomeruli from two different patients’ biopsies with Focal segmental glomerulosclerosis highlighting weak cytoplasmic staining of  TNFR2 in the podocytes. **F** Section from the corticomedullary junction highlights that TNFR2 expression was not identified by immunohistochemistry in collecting tubules and vasa-recta

**Fig. 5:**
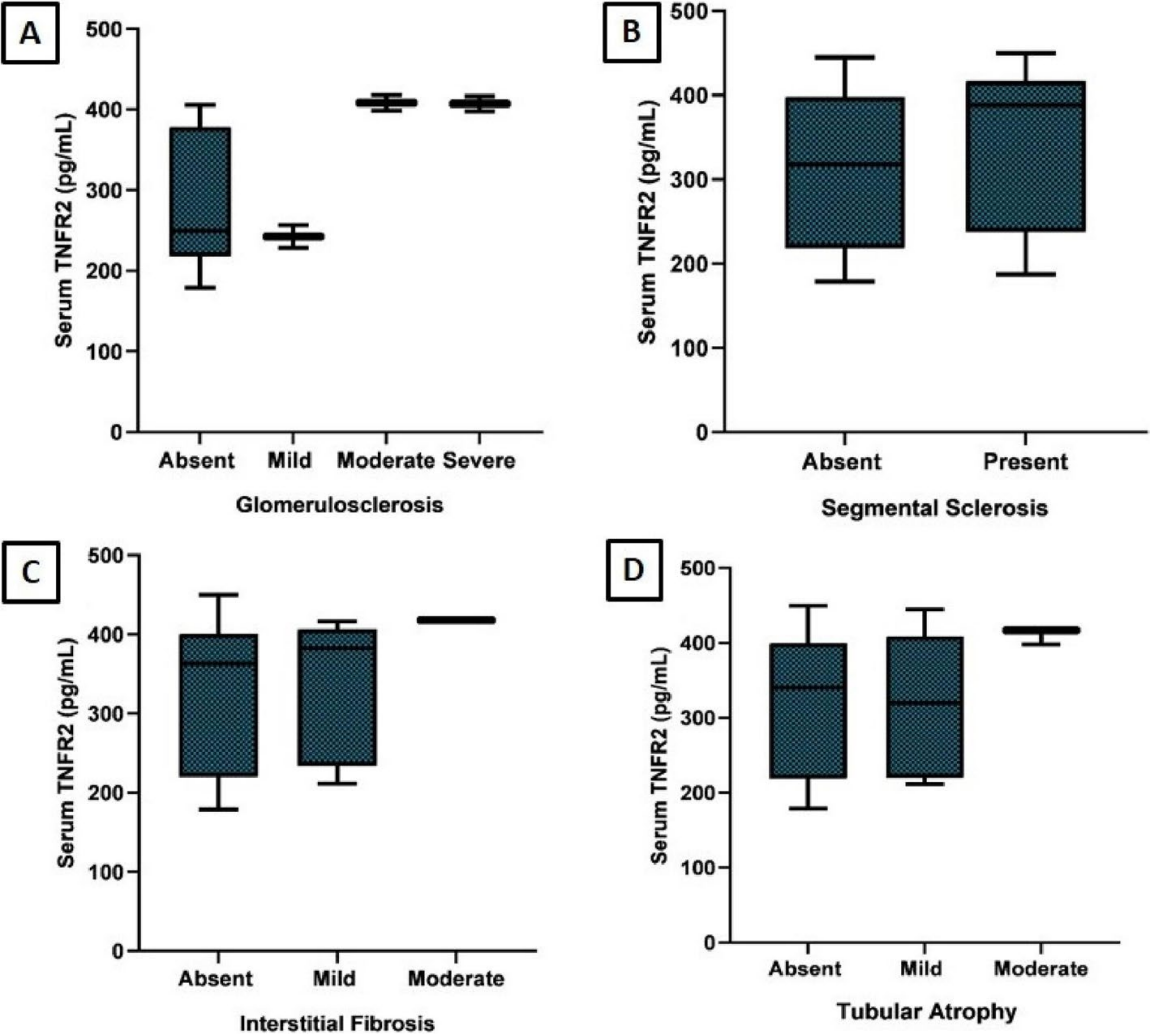
Histopathological assessment of renal abnormalities with serum TNFR2 levels in patients with podocytopathy. Histopathological assessment showing correlation between renal abnormalities and serum TNFR2 levels in patients with podocytopathy. This figure demonstrates how elevated TNFR2 levels correspond with histopathological changes, including glomerulosclerosis

**Fig. 6:**
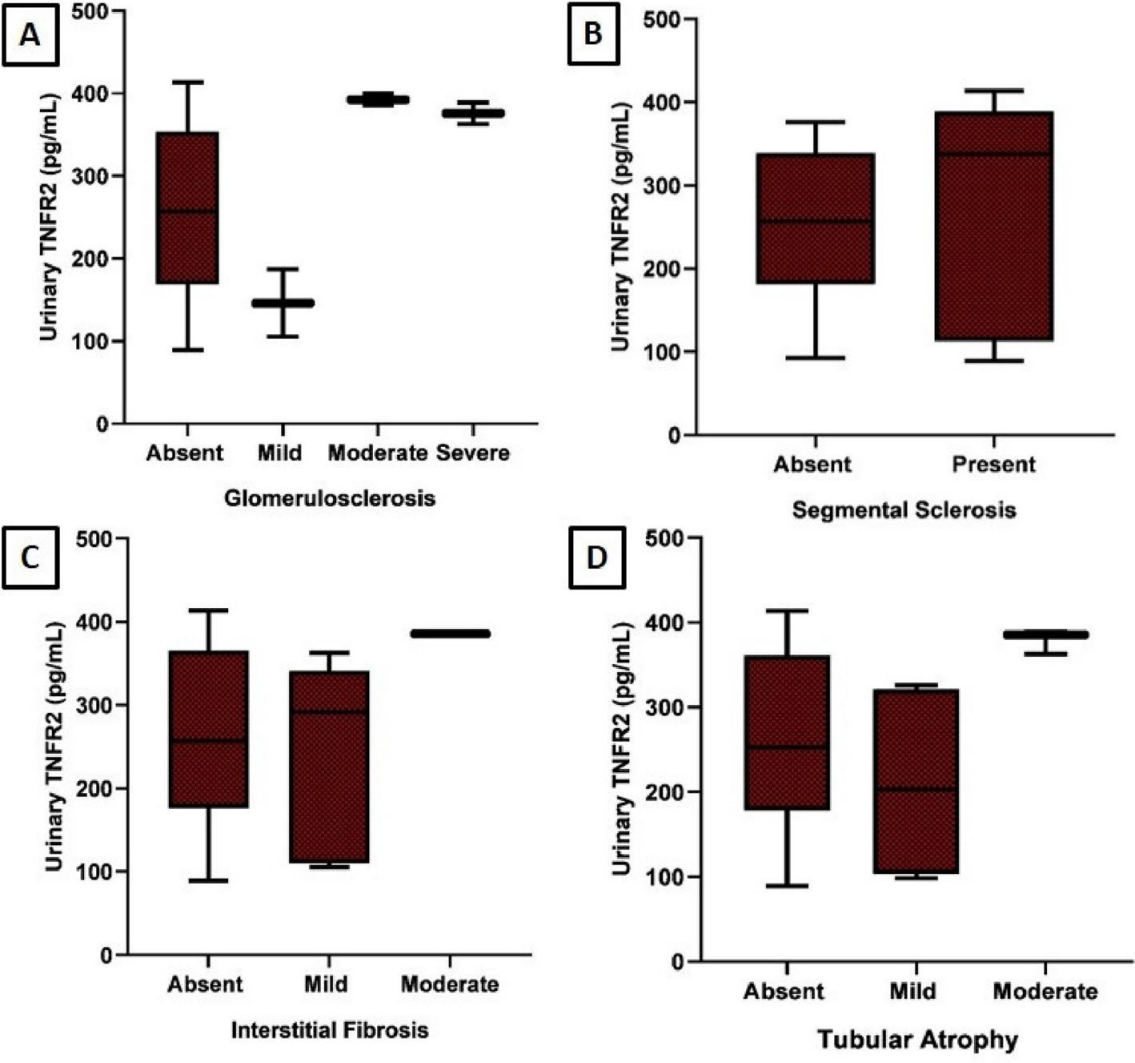
Histopathological assessment of renal abnormalities with urine TNFR2 levels in patients with podocytopathy. Histopathological assessment correlating renal abnormalities with urine TNFR2 levels in patients with podocytopathy. The figure illustrates the association between urine TNFR2 levels and histopathological chronicity scores, including glomerulosclerosis

The gene expression of TNFR2 was upregulated in patients with Podocytopathy, showing a 2.54-fold increase at baseline and a 2.29-fold increase at follow-up. TNFR2 gene expression was found to be statistically significant at baseline time point compared to healthy controls (*p* < 0.001) (Fig. 3A, B). TNFR2 immunohistochemical expression was assessed in 32 renal biopsies of patients with primary podocytopathy. TNFR2 was expressed in the PCT in 19 cases with a median H score 60 (IQR 40—120) and in the DCT with a median H score 45 (IQR 37.5—92.5) (Fig. 4A-D). TNFR2 expression in glomerular cells, including podocytes and mesangial cells, was also assessed. However, we found only focal weak staining in podocytes in the kidney biopsy restricted to 3 patients with FSGS precluding statistical analysis, while the expression was strongest in distal tubules followed by proximal tubules. Image shows the weak expression of TNFR2 in podocytes. (Fig. 4E, F) Serum TNFR2 levels at presentation were correlated with histopathological chronicity scores, showing statistical significance with glomerulosclerosis (*p* = 0.047) (Fig. 5A-D). Urine TNFR2 levels at presentation were also correlated with histopathological chronicity scores, demonstrating statistical significance with glomerulosclerosis (*p* = 0.026) (Fig. 6A-D). However, no statistically significant differences were found for segmental sclerosis, interstitial fibrosis, and tubular atrophy with serum and urine TNFR2 levels. No significant correlation was found between the H score in either the PCT or DCT with histopathological chronicity scores and eGFR at baseline and follow-up (Supplementary Table 1).

## Discussion

This study explored the prognostic implications of TNFR2 in patients with primary podocytopathy. Elevated serum TNFR2 levels were significantly associated with persistent eGFR decline, remission, and relapse in these patients, suggesting TNFR2’s potential as a biomarker for disease progression. Additionally, TNFR2 levels were positively correlated with proteinuria, indicating its involvement in renal damage. These findings support TNFR2 as a promising prognostic marker for assessing relapse, remission, and progression to stage 4 CKD in patients with podocytopathy.

Our findings revealed significantly elevated concentrations of TNF-α and TNFR2 in patients with podocytopathy compared to healthy controls. Previous studies have consistently shown that patients with FSGS exhibit notably higher levels of serum TNFR1, TNFR2, and TNF-α than healthy controls [6, 8]. This finding aligns with our results and emphasizes the role of TNFR2 as a key player in renal progression. In recent years, the role of inflammatory pathways, particularly the TNF-α pathway, has gained attention in the progression of CKD. TNFR2 has emerged as a promising biomarker with significant implications for the early diagnosis and prognosis of kidney diseases, such as MCD and FSGS. Our study highlights the potential of serum and urine TNFR2 levels with ELISA and RT-PCR as non-invasive biomarker for persistent eGFR decline and disease relapse, in correlation with urine protein and serum creatinine. This can potentially lead to earlier interventions and personalized treatment strategies. The role of serum and urine TNF and TNFR2 levels as biomarkers is critical due to the availability of several targeted anti-TNF drugs in immunological diseases, which needs further evaluation in renal disease.

Our approach, which utilizes highly sensitive and specific ELISA techniques for TNFR2 quantification, builds on existing methodologies while optimizing conditions for reproducibility and accuracy. Previous studies have demonstrated elevated TNFR2 levels in various kidney diseases [7, 9]. However, our work extends these findings by establishing a direct correlation between TNFR2 levels and long-term renal outcomes, including remission and relapse. The novel application of TNFR2 as a predictive biomarker for CKD progression underscores the relevance of this method in clinical settings and highlights the need for further exploration of TNF-α-targeted therapies to mitigate renal damage [20, 21].

Patients diagnosed with MCD also exhibited elevated serum levels of TNF-α and increased TNF-α secretion by monocytes compared to individuals in remission and control subjects [13]. Both mesangial cells and podocytes were found to increase TNF-α production through autocrine mechanisms, leading to the upregulation of TNFRs [22]. These elevated TNF pathway markers were observed to contribute significantly to renal impairment, with higher cTNFR levels in the greatest tertile correlating with a greater likelihood of renal progression [16].

In our current study, we observed a negative correlation between serum TNFR2 levels and the eGFR, indicating a potential link between TNFR2 expression and renal function decline. This observation aligns with previous findings from the NEPTUNE study group, which revealed a correlation between reduced eGFR and higher TNF expression in the glomeruli of few patients with FSGS, indicating a role for local TNF in the progression of glomerular diseases [8]. Gohda et al. also demonstrated a negative correlation between elevated serum TNFR levels with eGFR [7]. Additionally, increased levels of TNFR2 have been associated with a decline in renal function, further implicating the TNF-α inflammation pathway in disease progression. Notably, TNFR2 shows promise as an early predictor of CKD, as significantly higher levels are observed compared to the control group [9, 21].

Research conducted at the Joslin Diabetes Center supports these findings, indicating that elevated blood levels of cTNFRs can accurately predict an early decline in kidney function, leading to progression to CKD3 or ESRD in individuals with diabetic nephropathy [20, 23]. Numerous studies have highlighted the prognostic utility of TNFRs in various kidney conditions, including glomerulonephritis and IgA nephropathy, emphasizing their role as strong predictors of progressive kidney function impairment [20, 23–25]. In our research, we discovered that elevated serum TNFR2 levels were significantly associated with persistent decline in eGFR, remission, and relapse in patients with podocytopathy, indicating its potential as a biomarker. Moreover, TNFR2 was also found to be significant in the ROC analysis, further highlighting its prognostic utility.

We also noted increased urine TNFR2 levels and proteinuria in patients with MCD and FSGS. Interestingly, the serum and urine TNFR2 levels were positively correlated with the UPCR, although we did not find a correlation between the urine TNFR2 level and the eGFR. In line with our findings Gohda et al. reported a positive correlation between elevated serum TNFR levels and ACR [7]. This finding underscores the complex interplay between TNFR2 levels, proteinuria, and renal function in these conditions. Studies have shown that TNFR2 is not expressed in normal kidney tissue; however, in renal diseases, TNFR2 plays a critical, selective pro-inflammatory role in mediating renal injury [26]. Experimental studies have demonstrated that TNFR2 is expressed in the glomeruli in various glomerular diseases and in tubular epithelial cells during acute transplant rejection [27]. The expression of TNFRs in the human kidney suggests that the soluble form in the urine may partly arise from shedding by injured renal cells [28, 29]. In NS, this elevation may also result from the disruption of the glomerular filtration barrier, allowing larger molecules to pass into the urine, as well as enhanced cleavage and release of soluble TNFR2 due to inflammation. These mechanisms contribute to elevated urinary TNFR2 levels and reflect ongoing renal injury in glomerular diseases.

Podocytes, which are crucial for maintaining the glomerular filtration barrier, are susceptible to injury due to their delicate structure and limited regenerative capacity [30]. MCD has traditionally been linked to unknown circulating factors, potentially released by T cells directly targeting podocytes, leading to ultrastructural changes and proteinuria [31]. On the other hand, proteinuria is a hallmark of FSGS, resulting from podocyte injury caused by various factors such as circulating factors, gene mutations, infections, and diverse etiologies, culminating in massive proteinuria and histological changes in FSGS [32].

Studies have implicated the intrinsic podocyte TNF-α signaling pathway in the pathogenesis of FSGS. Treatment of isolated rat glomeruli with TNF-α increases albumin permeability through the generation of reactive oxidative species, while TNF-α infusion in mice leads to foot process effacement and the development of proteinuria [6]. These findings highlight the role of TNF-α and TNFR2 in podocyte injury and the pathophysiology of proteinuric kidney diseases.

We observed elevated TNFR2 gene and renal expression in individuals with podocytopathy. Mesangial cells showed moderate expression, particularly in areas adjacent to sclerotic lesions. We observed significantly higher TNFR2 expression in proximal and distal tubules in diseased kidneys. As was mentioned earlier, the mesangial and podocyte expression was restricted to 3 patients with FSGS and hence the same was not evaluated for statistical analysis. There was no expression of TNFR2 in collecting tubules. Specifically, when compared to controls and patients in remission, those with active MCD showed increased TNF-α mRNA expression in peripheral blood mononuclear cells (PBMCs) [13]. Lee et al. conducted a study in which patients with elevated cTNFR levels exhibited increased renal expression of TNFRs, as confirmed by immunohistochemical staining and real-time PCR. These findings suggest that kidney damage may contribute to the heightened expression of TNFRs in kidney disease [16]. In tubules characterized by atrophy and interstitial fibrosis, TNFR2 levels were particularly elevated, suggesting a role in the mechanisms of tubular injury and fibrosis. While our statistical analysis did not demonstrate significant associations, possibly due to limitations in sample size, but existing literature supports the biological relevance of our findings. Studies have also shown that TNFR2 renal expression increased with CKD severity and showed correlations with the score of mild and advanced tubular lesions and suggested that renal TNFR2 plays a role in CKD development, and has potential to be used as a biomarker for the early detection and progression of the disease [9]. Furthermore, our investigation revealed correlations between serum and urinary TNFR2 levels at presentation with glomerulosclerosis. These findings underscore the potential significance of TNFR2 in the pathogenesis of glomerular diseases and its association with disease severity and progression.

The high AUC for predicting progression to CKD stage 4 reflects the model’s ability to discriminate between patients who progressed to CKD stage 4 and those who did not. However, it’s important to note that the AUC from ROC analysis measures discrimination and does not directly incorporate time-to-event data, unlike the hazard ratio from Cox regression, which accounts for the timing of progression. The hazard ratio of 1.017 may seem modest, but it indicates that for every unit increase in TNFR2, the hazard (risk) of progressing to CKD stage 4 increases by 1.7%. Even small changes in hazard ratios can be meaningful, especially in chronic diseases where long-term risk accumulates. Additionally, the small sample size may limit the precision of our HR estimate, and it is possible that the significance of TNFR2 was underestimated due to this limitation. The sample size was relatively small, which may limit the generalizability of our findings. Despite these limitations, our results provide valuable insights into the prognostic utility of TNFR2 and its potential role in monitoring and managing podocytopathy.

## Conclusion

This research underscores the critical role of circulating TNFRs in kidney injury, particularly in primary podocytopathy. This study revealed that TNF pathway markers, specifically TNFR2, are significantly associated with renal impairment and an increased risk of renal progression. Notably, elevated serum TNFR2 levels emerged as a significant predictor of persistent decline in eGFR and disease relapse, that these levels could be used as biomarkers for disease progression and prognosis in individuals with primary podocytopathy. These results contribute valuable insights into the pathophysiology of kidney diseases and suggest avenues for targeted interventions to mitigate renal damage and improve patient outcomes.

## Supplementary information

Supplementary Material 1: Table 1. Correlation analysis for TNFR2 expression in renal biopsies with Histopathological chronicity scores

## Data Availability

Data will be available upon request to the corresponding author.

## Abbreviations

ACR: Albumin-to-creatinine ratio
CG: Collapsing glomerulopathy
CKD: Chronic kidney disease
cTNFR: Circulating tumor necrosis factor receptor
DCT: Distal convoluted tubules
eGFR: Estimated glomerular filtration rate
ELISA: Enzyme-linked immunosorbent assay
ESRD: End-stage renal disease
FSGS: Focal segmental glomerulosclerosis
GS: Glomerulosclerosis
IF: Interstitial fibrosis
MCD: Minimal change disease
NS: Nephrotic syndrome
PBMCs: Peripheral blood mononuclear cells
PCT: Proximal convoluted tubules
ROC: Receiver operating characteristic
TNF-α: Tumor necrosis factor-alpha
TNFR: Tumor necrosis factor receptor
UCPR: Urinary albumin-to-creatinine ratio
