# Supplementary material for: Role of baseline soluble tumor necrosis factor receptor 2 as a biomarker in primary podocytopathy: Implications for renal impairment and disease progression
Source: BMC Nephrol. 2024 Oct 25;25:378. doi: 10.1186/s12882-024-03772-y (PMC11515380; doi:10.1186/s12882-024-03772-y)
Supplement: Supplementary file 1 — Supplementary Material 1: Table 1. Correlation analysis for TNFR2 expression in renal biopsies with Histopathological chronicity scores [file 12882_2024_3772_MOESM1_ESM.docx]

**Table 1. Correlation analysis for TNFR2 expression in renal biopsies with Histopathological chronicity scores**

|  |  | **Glomerulosclerosis** | **Segmental sclerosis** | **Interstitial fibrosis** | **Tubular atrophy** |
| --- | --- | --- | --- | --- | --- |
| **PCT** | **r** | **0.071** | **-0.123** | **0.228** | **0.173** |
|  | p -value | **0.726** | **0.540** | **0.253** | **0.387** |
| **DCT** | **r** | **-0.230** | **-0.269** | **0.149** | **0.133** |
|  | p -value | **0.910** | **0.175** | **0.457** | **0.510** |
